# Subsequent SARS‐CoV‐2 Infection in Healthcare Workers: A Retrospective Cohort Study in Iran

**DOI:** 10.1002/iid3.70355

**Published:** 2026-03-18

**Authors:** Yeganeh Karimi, Arash Abdollahi, Seyyed Amir Yasin Ahmadi, Taghi Riahi, Saeed Kalantari, Nahid Nafissi, Sima Shokri, Hamid Reza Baradaran, Azadeh Goodarzi

**Affiliations:** ^1^ Faculty of Medicine Iran University of Medical Sciences (IUMS) Tehran Iran; ^2^ Department of Epidemiology, School of Public Health Iran University of Medical Sciences Tehran Iran; ^3^ Minimally Invasive Surgery Research Center Iran University of Medical Sciences Tehran Iran; ^4^ Preventive Medicine and Public Health Research Center, Psychosocial Health Research Institute Iran University of Medical Sciences (IUMS) Tehran Iran; ^5^ Department of Internal Medicine, School of Medicine, Rasool Akram Medical Complex Iran University of Medical Sciences Tehran Iran; ^6^ Antimicrobial Resistance Research Center, Institute of Immunology and Infectious Diseases Iran University of Medical Sciences Tehran Iran; ^7^ Department of General Surgery, Rasool Akram Medical Complex Clinical Research Development Center (RCRDC) Iran University of Medical Sciences Tehran Iran; ^8^ Department of Allergy and Clinical Immunology, Rasool Akram Medical Complex Clinical Research Development Center (RCRDC), School of Medicine Iran University of Medical Sciences Tehran Iran; ^9^ Aging Clinical and Experimental Research Team, Institute of Applied Health Sciences, School of Medicine, Medical Sciences and Nutrition University of Aberdeen Aberdeen UK; ^10^ Department of Dermatology, Rasool Akram Medical Complex Clinical Research Development Center (RCRDC), School of Medicine Iran University of Medical Sciences Tehran Iran

**Keywords:** COVID‐19, healthcare workers, occupational risk, pandemic, public health, reinfection, risk factors, SARS‐CoV‐2, vaccination

## Abstract

**Background:**

The evolution of SARS‐CoV‐2 has increased global COVID‐19 case prevalence. Healthcare workers (HCWs) are particularly susceptible to reinfection due to their exposure to infected patients. This study aimed to explore the risk factors for subsequent SARS‐CoV‐2 infection among HCWs.

**Methods:**

This retrospective study was conducted in a teaching hospital affiliated with the Iran University of Medical Sciences between March 2021 and October 2021. HCWs completed an online survey to gather information about their COVID‐19 infection history. Subsequent SARS‐CoV‐2 infection was defined as two or more infections, regardless of the time frame interval. Individuals who experienced a second infection within 90 days of the initial infection were considered to have a recurrence, and others were considered to have a reinfection. The generalized estimation equation was utilized to develop a multivariable prediction model.

**Results:**

A total of 601 HCWs (60.2% female), mostly aged 18–29 years (47.4%), participated in this study. We observed 90 episodes of subsequent SARS‐CoV‐2 infection among HCWs, including 72 individuals (11.98%). Among them, 9 participants (1.5%) experienced recurrence, while 43 participants (7.1%) reported reinfection. In the multivariable analysis, vaccination significantly reduced the risk of subsequent SARS‐CoV‐2 infection, whereas nurses and those working in COVID‐19 wards faced twice the risk.

**Conclusion:**

The frequency of subsequent SARS‐CoV‐2 infection is significant among HCWs. Vaccination status, occupation type, and workplace are key factors influencing the risk of future infections.

## Summary

- What is known about this topic
- SARS‐CoV‐2 mutations contribute to reinfections, prolonging the pandemic. However, the role of exposure in the reinfection of healthcare workers has been controversial.
- What this article adds
- Vaccination significantly reduced the risk of subsequent SARS‐CoV‐2 infection. Increased exposure to COVID‐19 cases, such as being a nurse and working in a COVID‐19 ward, increases the risk of subsequent infection.

## 1 Introduction

Since the emergence of severe acute respiratory syndrome coronavirus 2 (SARS‐CoV‐2), it was assumed that recovery from coronavirus disease 2019 (COVID‐19) would confer lasting immunity. However, the virus's high mutation rate has generated region‐specific variants that reduce immune recognition [1], while antibody titers diminish over time [2]. The first confirmed reinfection, reported in a 33‐year‐old man in Hong Kong, involved a genetically distinct variant 142 days after the initial infection, despite interim negative tests [3]. Persistent genetic evolution of SARS‐CoV‐2 continues to generate new variants that alter transmissibility, immune escape, and clinical severity, challenging ongoing efforts in diagnosis, treatment, vaccination, and public‐health control [4].

Studying reinfection clarifies the durability of immunity from natural infection and vaccination, informing the need for booster doses and evaluation of vaccine effectiveness [5, 6]. It also guides evidence‐based public health decisions, such as vaccine distribution and preventive measures, and helps assess how natural and vaccine‐induced immunity interact in achieving herd immunity.

Reinfection risk depends on several factors, including vaccination status, variant type, pandemic wave, initial infection severity, comorbidities, and age [6, 7, 8, 9]. Yet, healthcare workers (HCWs) exhibit infection rates about three times higher than the general population [10], with recent studies reporting reinfection rates of 5.9%–20.6%, particularly during the Omicron period [7, 11, 12, 13, 14, 15]. Previous studies suggest that occupational exposure, work environment, vaccination status, age, sex, clinical role, and comorbidities may influence reinfection risk among HCWs [5, 6, 7, 11, 16, 17].

Given HCWs' susceptibility to COVID‐19, this study aims to assess the frequency of subsequent self‐reported SARS‐CoV‐2 infection among HCWs and to explore potential risk factors associated with a higher likelihood of reinfection.

## 2 Methods

### 2.1 Study Design and Participants

This retrospective study, conducted from March 2021 to October 2021, used a structured online survey to assess the frequency of subsequent SARS‐CoV‐2 infections since the beginning of the pandemic and to identify potential risk factors among HCWs. We invited all HCWs employed at the Hazrat Rasool Medical Center, a referral teaching hospital (800 beds) affiliated with Iran University of Medical Sciences, during the COVID‐19 pandemic. Exclusion criteria included individuals who declined to provide informed consent, those with incomplete online surveys, or those who could not recall specific details of their COVID‐19 history.

This study received approval from the Iran University of Medical Sciences Ethics Committee (IR.IUMS.FMD.REC.1401.034). Informed consent was obtained from all participants before their engagement in the study.

### 2.2 Data Collection and Online Survey Development

A team of specialists created a structured online survey based on established guidelines and scholarly references. This comprehensive online survey included several sections: demographics, SARS‐CoV‐2 vaccination status, prior COVID‐19 diagnoses, medical history, personal protective equipment (PPE) use, and familial COVID‐19 history. Additionally, specific queries were integrated to evaluate symptoms associated with the initial and potential subsequent episodes. All questions employed a multiple‐choice format to mitigate response errors. The online survey was administered electronically via a secure online platform using text messages and various social media channels. It was recurrently shared within social media groups to maximize the number of respondents.

### 2.3 Study Variables and Outcomes

Participant identities were protected by assigning unique ID codes, and data were analyzed anonymously. Key study variables included age, sex, education, blood group, past medical history, smoking and alcohol consumption habits, occupation, workplace, weekly duration of employment, use of PPE, family history of COVID‐19, and vaccination status. The study outcome was defined as a subsequent SARS‐CoV‐2 infection. Because of the potential recall bias in estimating the time between two infection episodes, we considered a subsequent SARS‐CoV‐2 infection, rather than true reinfection, as the primary outcome.

### 2.4 COVID‐19 Diagnosis and Definitions

COVID‐19 diagnoses were established based on a physician's assessment, using chest CT scans and/or polymerase chain reaction (PCR) tests on nasopharyngeal or oropharyngeal samples, based on participants' self‐reported information. “Subsequent SARS‐CoV‐2 infection” was defined as the occurrence of two or more SARS‐CoV‐2 infections, regardless of the time interval between the episodes [18, 19, 20]. If the second infection occurred within 90 days of the first, it was classified as a “recurrence”; otherwise, it was classified as a “reinfection” [21]. All patients were categorized into two groups based on subsequent SARS‐CoV‐2 infection. Participants who smoked cigarettes were categorized as light (less than 20 pack‐years), moderate (20–40 pack‐years), and heavy (≥ 40 pack‐years) smokers [22]. Individuals were classified into four occupational groups—nurses, physicians, clinical staff, and nonclinical staff—based on their exposure to COVID‐19 cases. To overcome the overlap between occupational categories, every participant (except physicians and nurses) who worked in the clinical ward, regardless of other roles in non‐clinical wards, was categorized as clinical staff and remained classified as non‐clinical. Physicians consisted of specialists, fellows, residents, and medical interns. Clinical staff comprised individuals working in clinical wards or engaging in direct patient interactions, including clinical staff and supervisors, ward secretaries, clinical services staff, paraclinical technologists, nurse aides, and medical trainees. Nonclinical staff encompassed administrative staff, administrative supervisors, administrative services staff, and medical students.

### 2.5 Statistical Analysis

The data set was organized longitudinally according to vaccination status, encompassing three time periods: before vaccination, after the first dose, and after the second dose. Data on SARS‐CoV‐2 infection and subsequent infection episodes were recorded as repeated measures across these stages: before vaccination, after the first dose, and after the second dose. Descriptive statistics were employed to present study variables as frequencies (percentages), and *χ* ^2^ and Fisher's exact tests were utilized for analysis. Subsequently, a multivariable generalized estimation equation (GEE), a logistic regression model, was implemented to identify potential risk factors for subsequent SARS‐CoV‐2 infection. Vaccination status was treated as a within‐subject variable with an autoregressive order 1 (AR1) correlation structure. Variables were selected backward, retaining those with *p* values ≤ 0.1 in the multivariable model. Data analysis was performed using IBM SPSS Statistics version 26 and STATA software version 17 (StataCorp. LLC, US).

### 2.6 Sources of Bias

In this cohort study, the researchers did not control for vaccination status, and participants were not randomized concerning this factor, which could lead to selection bias. Furthermore, relying on self‐reported data may introduce recall bias.

## 3 Results

A total of 601 HCWs were included in this study, and their baseline characteristics are summarized in Table 1. The majority of participants were female (60.2%), with approximately half of them aged 18–29 years (47.4%). Among the participants, 338 (56.2%) reported no underlying medical conditions. Comorbidities were distributed as follows: 14 participants (2.3%) had diabetes mellitus, 23 participants (3.8%) had hypertension, 9 participants (1.5%) had cardiovascular disorders, 31 participants (5.2%) had asthma, and four participants (0.7%) had chronic obstructive pulmonary disease. Current smokers accounted for 33 individuals (5.5%), and all of them were light smokers (less than 20 pack‐years). Regarding occupation, 35.1% were physicians, 19.6% were nurses, while the remaining participants (45.3%) encompassed clinical and nonclinical staff members and students. Additionally, 8.2% of participants were employed in COVID‐19 wards. Regarding vaccination status, 17 participants (2.8%) had not received any vaccines, while 20 participants (3.3%) had received only the first dose. The most frequently administered vaccines were Sputnik (36.8%) and AstraZeneca (30.4%). Other vaccines included Sinopharm, Pfizer, Bharat, Barkat, and Razi. Notably, 90 episodes of subsequent SARS‐CoV‐2 infection were detected (15.0%), involving 72 individuals (11.98%). Among them, 9 participants (1.5%) experienced a recurrence, while 43 participants (7.1%) reported reinfection. Specifically, 54 participants (9.0%) experienced subsequent SARS‐CoV‐2 infection before vaccination, eight participants (1.4%) after the first vaccine dose, and 28 participants (5.0%) after the second dose. Significant differences were observed in occupation categories (*p* = 0.03) and types of masks used (*p* = 0.01) between patients with subsequent SARS‐CoV‐2 infection and those without. Additionally, the type of blood group, smoking status, alcohol use, work duration, usage of gloves, eye protection, or gown, familial history of COVID‐19, vaccination status, and vaccine brand, and working in the COVID‐19 ward were not significantly different between the two groups in the univariate analysis.

**Table 1:** Baseline characteristics of the study participants according to subsequent SARS‐CoV‐2 infection.

Characteristics^a^	Total (*n* = 601)	Participants with subsequent infection (*n* = 72, 11.98%)	Participants without subsequent infection (*n* = 529, 88.02%)	*p* value^b^
Age group				0.88
18–29 years	285 (47.4)	35 (48.6)	250 (47.3)	
30–39 years	153 (25.5)	17 (23.6)	136 (25.7)	
40–49 years	109 (18.1)	14 (19.4)	95 (18.0)	
50–59 years	47 (7.8)	6 (8.3)	41 (7.8)	
≥ 60 years	7 (1.2)	0 (0)	7 (1.3)	
Sex				0.72
Male	239 (39.8)	30 (41.7)	209 (39.5)	
Female	362 (60.2)	42 (58.3)	320 (60.5)	
Blood group				0.43
A+	187 (31.1)	25 (34.7)	162 (30.6)	
A−	18 (3)	3 (4.2)	15 (2.8)	
B+	112 (18.6)	10 (13.9)	102 (19.3)	
B−	14 (2.3)	2 (2.8)	12 (2.3)	
O+	181 (30.1)	24 (33.3)	157 (29.7)	
O−	28 (4.7)	2 (2.8)	26 (4.9)	
AB+	44 (7.3)	2 (2.8)	42 (7.9)	
AB−	6 (1)	2 (2.8)	4 (0.8)	
Medical condition				
No underlying disease	338 (56.2)	39 (54.2)	299 (56.5)	0.70
Diabetes mellitus	14 (2.3)	3 (4.2)	11 (2.1)	0.27
Hypertension	23 (3.8)	2 (2.8)	21 (4.0)	1.00
Cardiovascular disorders	9 (1.5)	1 (1.4)	8 (1.5)	1.00
Asthma	31 (5.2)	4 (5.6)	27 (5.1)	0.78
Chronic obstructive pulmonary disease	4 (0.7)	1 (1.4)	3 (0.6)	0.40
Smoker	33 (5.5)	2 (2.8)	31 (5.9)	0.14
Alcohol use	21 (3.5)	2 (2.8)	19 (3.6)	1.00
< 14 g daily	19 (3.2)	1 (3.6)	18 (3.1)	0.99
14–28 g daily	1 (0.2)	0 (0)	1 (0.2)	
> 28 g daily	1 (0.2)	0 (0)	1 (0.2)	
Occupation				0.03
Physician	211 (35.1)	19 (26.4)	192 (36.3)	
Nurse	118 (19.6)	23 (31.9)	95 (18.0)	
Clinical staff	212 (35.3)	25 (34.7)	187 (35.3)	
Non‐clinical staff	60 (10)	5 (6.9)	55 (10.4)	
Work in clinical wards				0.15
Covid‐19 ward	49 (8.2)	9 (12.5)	40 (7.6)	
Non‐covid‐19 ward	428 (71.2)	53 (73.6)	375 (70.9)	
Work duration, weekly				0.57
< 30 h	148 (24.6)	14 (19.4)	134 (25.3)	
30–50 h	271 (45.1)	32 (44.4)	239 (45.2)	
> 50 h	165 (27.5)	23 (31.9)	142 (26.8)	
Education				0.12
Student	162 (27)	22 (30.6)	140 (26.5)	
Diploma and under‐diploma	49 (8.2)	8 (11.1)	41 (7.8)	
Upper‐diploma, BSc, MSc	196 (32.6)	27 (37.5)	169 (31.9)	
GP, PhD, and higher levels	189 (31.4)	14 (19.4)	175 (33.1)	
Personal protective equipment (PPE)				
Kind of mask				0.01
Cloth mask	13 (2.2)	2 (2.8)	11 (2.1)	
Surgery mask	200 (33.3)	28 (38.9)	172 (32.5)	
Two surgery masks	97 (16.1)	9 (12.5)	88 (16.6)	
N95	96 (16)	17 (23.6)	79 (14.9)	
N95+ surgery mask	54 (9)	10 (13.9)	44 (8.3)	
Gloves				0.34
None	139 (23.1)	23 (31.9)	116 (21.9)	
Latex	317 (52.7)	34 (47.2)	283 (53.5)	
Plastic	52 (8.7)	4 (5.6)	48 (9.1)	
Both	33 (5.5)	3 (4.2)	30 (5.7)	
Eye protection				0.67
None	328 (54.6)	36 (50.0)	292 (55.2)	
Glass	83 (13.8)	9 (12.5)	74 (14.0)	
Shield	124 (20.6)	16 (22.2)	108 (20.4)	
Both	19 (3.2)	4 (5.6)	15 (2.8)	
Gown	252 (41.9)	33 (45.8)	219 (41.4)	0.41
Family history				
Hospitalization of relatives	125 (20.8)	17 (23.6)	108 (20.4)	0.82
Death of relatives	76 (12.6)	12 (16.7)	64 (12.1)	0.54
COVID‐19 vaccination	584 (97.2)	3 (4.2)	14 (2.6)	0.44
Vaccine brand				0.90
No vaccine	17 (2.8)	3 (4.2)	14 (2.6)	
Sputnik	221 (36.8)	28 (38.9)	193 (36.5)	
Sinopharm	102 (17)	13 (18.0)	89 (16.8)	
Astrazeneca	183 (30.4)	20 (27.8)	163 (30.8)	
Other^c^	78 (13)	8 (11.1)	70 (13.3)	

Abbreviations: BSc, bachelor of science; GP, general practitioner; MSc, master of science; PhD, doctor of philosophy.

^a^ Categorical variables are presented as numbers (%).

^b^ *p* ≤ 0.05 is considered significant.

^c^ Pfizer, Razi, Bharat, and Barkat.

**Figure 1:**
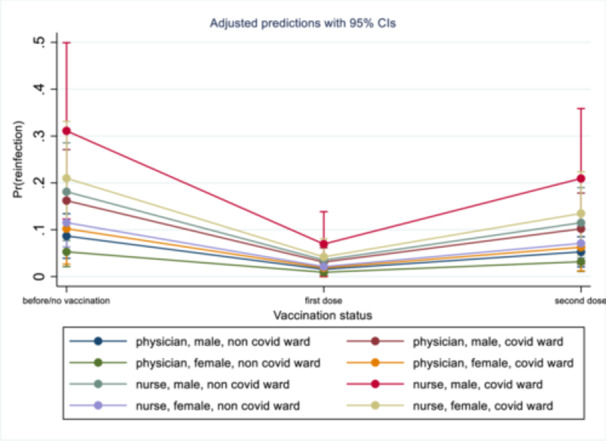
Prediction model for subsequent SARS‐CoV‐2 infection based on vaccination status. The model was developed using a generalized estimating equation (GEE).

The unadjusted GEE analysis showed that the risk of subsequent SARS‐CoV‐2 infection decreased in HCWs vaccinated with one (OR, 0.14, 95% CI, 0.07–0.27; *p* < 0.001) or two doses (OR, 0.49, 95% CI, 0.31–0.78; *p* < 0.01) while being a nurse (OR, 2.17, 95% CI, 1.19–3.97; *p* = 0.01) was an independent risk factor for subsequent SARS‐CoV‐2 infection. After a multivariable adjustment, being a nurse (OR, 2.33, 95% CI, 1.15–4.74; *p* = 0.02) and working in the COVID‐19 ward (OR, 2.04, 95% CI, 1.04–4.02; *p* = 0.04) were independent risk factors for subsequent SARS‐CoV‐2 infection and vaccination with one (OR, 0.16, 95% CI, 0.08–0.32; *p* < 0.001) or two doses (OR, 0.59, 95% CI, 0.36–0.95; *p *= 0.03) significantly reduced the risk (see Table 2 and Figure 1).

**Table 2:** Prediction of Subsequent SARS‐CoV‐2 infection, generalized estimation equation multivariable analysis.

Variables	Unadjusted		Adjusted	
	OR (95% CI)	*p* value^a^	OR (95% CI)	*p* value^a^
Vaccination status				
Before vaccination	Ref	Ref	Ref	Ref
After the first dose	0.14 (0.07–0.27)	< 0.001	0.16 (0.08–0.32)	< 0.001
After the second dose	0.49 (0.31–0.78)	< 0.01	0.59 (0.36–0.95)	0.03
Gender				
Female	Ref	Ref	Ref	Ref
Male	1.18 (0.75–1.86)	0.48	1.70 (0.96–3.02)	0.07
Occupation				
Physician	1.00	1.00	1.00	1.00
Nurse	2.17 (1.19–3.97)	0.01	2.33 (1.15–4.74)	0.02
Clinical staff	1.25 (0.69–2.24)	0.46	1.16 (0.60–2.24)	0.66
Non‐clinical staff	1.44 (0.47–2.78)	0.76	0.83 (0.24–2.87)	0.77
Workplace				
Non covid‐19 ward	Ref	Ref	Ref	Ref
Covid‐19 ward	1.83 (0.94–3.58)	0.08	2.04 (1.04–4.02)	0.04

Abbreviations: 95% CI, 95% confidence interval; OR, odds ratio; ref, reference.

^a^ *p* ≤ 0.05 considered significant.

## 4 Discussion

Our findings demonstrate that occupational exposure plays a key role in subsequent SARS‐CoV‐2 infection among HCWs, particularly for those working in COVID‐19 wards and nurses who experience more direct and prolonged contact with infected patients. Baseline factors such as vaccination status and sex may also contribute to susceptibility.

Our study identified a 15% frequency of subsequent SARS‐CoV‐2 infection, indicating a considerable rate among healthcare workers in Iran. Reported subsequent infection rates among HCWs in previous studies have varied widely, largely due to differences in diagnostic certainty, inclusion of probable cases, and the criteria used to define recurrence or reinfection based on the time interval between positive SARS‐CoV‐2 tests [13, 23, 24, 25, 26]. For instance, reinfection and recurrence rates were reported as 5.94% and 20.57%, respectively, among HCWs in the United States in 2020 [7]. In Iran, a 2021 study found a reinfection rate of 6.3% among seronegative HCWs during the country's third wave [27], while another study reported a 13.7% reinfection rate at a similar stage of the pandemic [28]. Unlike earlier studies, our analysis, with a relatively higher frequency of subsequent infection, spans five pandemic waves, offering a broader view of reinfection dynamics across different phases of viral transmission and the vaccination rollout in Iran. Our findings indicate that despite vaccination and the end of the pandemic, HCWs are still at risk of subsequent infection, particularly with the latest variant. This underscores the importance of preventive measures, especially for those at higher risk of severe infection.

Most infection episodes in our cohort occurred before vaccination, underscoring the protective role of immunization. The frequency of subsequent infection declined from 9.0% before vaccination to 1.4% after the first dose and 5.0% after the second dose, likely reflecting reduced immunity over time. This trend is consistent with previous studies showing that vaccination markedly reduces the risk of reinfection. Hall et al. observed a sharp decline in reinfection rates after vaccination [29], and other studies similarly confirmed lower reinfection among fully vaccinated individuals [6, 30]. Overall, these findings reinforce the effectiveness of COVID‐19 vaccines and highlight the importance of booster doses in maintaining protection.

Our study found that occupational exposure strongly influenced reinfection risk among HCWs. Nurses had more than twice the likelihood of subsequent infection compared with physicians and other staff, and working in a COVID‐19 ward similarly doubled the risk relative to non‐COVID‐19 units. These results emphasize that frequent and direct contact with infected patients substantially increases the risk of reinfection. Although one study found no difference in reinfection across occupational groups [31], several other studies reported a higher risk among HCWs with close patient contact, including nurses and physicians [5, 26, 32]. The consistency of these findings across studies reinforces the link between exposure intensity and reinfection risk observed in our cohort.

Our study found no significant association between subsequent SARS‐CoV‐2 infection and demographic or lifestyle factors, including age, blood group, comorbidities, smoking, alcohol use, family history of COVID‐19, or education. Mask type and PPE use also showed no independent relationship with subsequent infection after adjustment for confounders. These results are consistent with several studies reporting similar findings [28, 32, 33], although others have suggested possible links between reinfection and factors such as older age, immunosuppression, or comorbidities [6, 9, 28, 32]. Differences in design, population, and infection control practices may explain these inconsistencies. The impact of PPE remains uncertain and may depend on correct use and training. As previous reviews highlight, proper PPE use substantially reduces viral transmission [34], yet this study could not assess transmissibility due to unmeasured asymptomatic infections. Notably, the type of masks used by HCWs was significantly different between individuals with subsequent infection and those without. Future research should include data on PPE adherence, training, and asymptomatic cases to clarify their influence on reinfection risk.

This study has several limitations. Its single‐center design may limit generalizability to other regions with different healthcare settings, vaccination policies, or transmission dynamics. Reliance on self‐reported infections introduces potential recall bias and diagnostic uncertainty, particularly in cases without confirmatory PCR testing; however, this approach allowed inclusion of a larger and more diverse group of healthcare workers, improving representativeness. The voluntary nature of participation may have introduced selection bias, and asymptomatic reinfections were likely underdetected due to the absence of routine PCR screening, preventing assessment of viral transmissibility. Moreover, data collection occurred before the emergence of the Omicron variant, whose distinct transmission and reinfection patterns limit the applicability of these findings to later pandemic phases. Despite these limitations, the study's strengths include a comprehensive data collection encompassing demographic, vaccination, occupational, and workplace variables, a diverse hospital‐wide sample, and robust analysis using a GEE model. Future studies should use multicenter prospective designs with genomic confirmation and systematic testing to validate and extend these findings across different healthcare contexts and viral variants.

## 5 Conclusion

In conclusion, our study underscores the heightened risk of subsequent SARS‐CoV‐2 infection among male nurses and those working in COVID‐19 wards due to intense interpersonal contact with infected cases. However, vaccination significantly reduced the risk. Notably, vaccines are more effective in preventing severe COVID‐19 [8], while non‐pharmaceutical risk reduction measures (like PPE) are the most effective tools to contain the spread of SARS‐CoV‐2 in healthcare settings [35]. These findings support targeted interventions, such as assigning high‐risk individuals to non‐COVID wards, enhancing training on the proper use of PPE, and considering booster vaccinations to better protect HCWs. Future prospective studies with long‐term follow‐up should evaluate the durability of vaccine‐induced immunity and the effectiveness of different booster dose schedules in high‐risk HCWs. Additionally, long‐term health outcomes, including COVID‐19 complications, and the role of SARS‐CoV‐2 variants in reinfection rates should be considered.

## Author Contributions

Y.K. contributed significantly to the study design, questionnaire development, data acquisition, data production, analysis, and data interpretation and took the lead in writing the initial and final drafts of the manuscript. A.A. played a role in data acquisition, data production, and analysis, and contributed to the initial draft of the manuscript. S.A.Y.A. analyzed and interpreted the data set. T.R., S.K., N.N., and S.S. facilitated questionnaire distribution and data acquisition. H.R.B. played a crucial role in shaping the study design, questionnaire development, analysis, interpretation, and revision of the manuscript. A.G. played a significant role in shaping the study design, questionnaire development, data acquisition, data production, and manuscript revision. All the authors have read and approved the final manuscript.

## Funding

The authors received no specific funding for this work.

## Ethics Statement

This study received approval from the Iran University of Medical Sciences Ethics Committee (IR.IUMS.FMD.REC.1401.034).

## Consent

Informed consent was obtained from all participants before their involvement in the study.

## Conflicts of Interest

The authors declare no conflicts of interest.

## Data Availability

The data sets generated and/or analyzed during the current study are not publicly available due to participant confidentiality policies and laws, but are available from the corresponding author upon reasonable request.
